# Quantification of FDG in the spinal cord using PET/MRI

**DOI:** 10.3389/fnume.2025.1646662

**Published:** 2025-08-26

**Authors:** Eve Lennie, Steven Sourbron, Nigel Hoggard, Thomas Jenkins, Charalampos Tsoumpas

**Affiliations:** ^1^ Division of Clinical Medicine, University of Sheffield, Sheffield, United Kingdom; ^2^ Department of Radiology, Sheffield Teaching Hospitals NHS Foundation Trust, Sheffield, United Kingdom; ^3^ Joondalup Healthcare Campus and Curtin University, Perth, WA, Australia; ^4^ Department of Nuclear Medicine and Molecular Imaging, University Medical Center Groningen, University of Groningen, Groningen, Netherlands

**Keywords:** PET/MRI, positron emission tomography, magnetic resonance imaging, spinal cord, neurology, neuroimaging, quantification

## Abstract

**Background:**

In this study, we investigate the impact of MR-derived attenuation maps and limited detector resolution on the quantification of positron emission tomography (PET) activity uptake in the spinal cord during PET/MRI. This was performed by simulating [${}^{18}$F]FDG PET data in the neck and thorax and then modifying the attenuation map to remove bone features. We then compared Ordered Subset Expectation Maximisation-reconstructed images to those with full attenuation correction. This simulation was performed at two detector resolutions of 2.1 and 4.4 mm. Acquisitions from a clinical study were then used to assess the ability of point spread function (PSF) modelling and time-of-flight (TOF) corrections, as implemented on the SIGNA PET/MR scanner (GE HealthCare), to correct for these quantification errors. For comparison, mean uptake was measured in regions of interest at each vertebral position along the spinal cord.

**Results:**

Simulation results showed a decreasing pattern of uptake from the cervical to the thoracic spinal cord. When bone was not included in attenuation correction, the mean uptake decreased by 3%–10.4%. This difference in measured uptake was 6.4%–23.9% in images simulated at a detector resolution representative of a clinical PET/MRI scanner. At a detector resolution of 4.4 mm, a 32.2% decrease in uptake was measured compared to the 2.1 mm simulation. In patient data, introducing vertebral bone to the attenuation correction pseudo-CT led to a 1.8%–18.3% difference in ${\mathrm{SUV}}_{\mathrm{mean}}$ in the spinal cord. Applying PSF modelling did not lead to any statistically significant changes. TOF correction reduces the difference in ${\mathrm{SUV}}_{\mathrm{mean}}$ between data attenuation corrected with and without vertebral bone to 4.3%–7%. TOF Q.Clear images with beta = 100 showed the smallest difference between attenuation correction approaches at 0.6%–5.2%.

**Conclusion:**

Ignoring bone during image reconstruction in PET/MRI reduces the activity measured during quantification of the spinal cord; however, the partial volume effect has a greater impact on reducing measured uptake in lower-resolution data. While time-of-flight correction goes somewhat resolves these quantification errors, further research is needed into partial volume correction.

## 1 Introduction

Combined positron emission tomography and magnetic resonance imaging (PET/MRI) has been used extensively in the study of neurological conditions in the brain; however, there are no reliable biomarkers in the spinal cord for a number of neurodegenerative diseases (1). Previous studies on spinal cord PET and PET with computed tomography (PET/CT) in adults have observed a generally decreasing pattern of [${}^{18}$F]FDG uptake in the spinal cord from the cervical to the thoracic spine, with a peak in the lower cervical spine (2–4). This has been explained as being due to an increased amount of grey matter and an enlargement of the spinal cord diameter (2). However, partial volume effects may lead to an underestimation of activity in other regions of the spinal cord with a smaller (<10 mm) size (3). Additionally, it is reported that there are quantitative differences in spinal cord uptake between PET/CT and PET/MRI (5). One explanation is that bone properties are not accounted for in attenuation and scatter correction in PET/MRI when using attenuation maps derived from a Dixon MRI sequence. Some brain imaging protocols implement a zero echo time (ZTE) or ultrashort echo time (UTE) sequence (6, 7), which allows for the skull to be delineated and assigned bone attenuation values. However, no commercially available software has implemented solutions for other areas of the body (8). Some attempts to resolve attenuation correction challenges have lead to investigations into deep learning (9) and other novel methods such as dual tracer imaging to implement an additional bone tissue class into pseudo-CT generation (10).

The first aim of this study is to investigate whether failing to fully account for the attenuation and scatter contributions of bone during image reconstruction leads to errors in measured spinal cord activity. This is achieved through a simulation study using the XCAT mathematical phantom (11) to simulate an [${}^{18}$F]FDG PET activity distribution that includes the spinal cord, which allows for simulated MR-derived attenuation maps to be compared to a known true attenuation map. We also investigate how this is impacted by detector resolution and partial volume effects.

The second aim is to determine whether currently implemented image reconstruction methods can resolve attenuation correction errors and partial volume effects in the spinal cord through the use of point spread function (PSF) modelling and time-of-flight (TOF) corrections. The use of TOF image reconstruction algorithms has previously been reported to reduce quantification errors in bones and lungs when MR-derived attenuation maps are used (12) and to improve image contrast and the detection of small lesions (13). Further substantial improvements in TOF might even eliminate the need for PET image reconstruction (14). We compared mean standardised uptake values (${\mathrm{SUV}}_{\mathrm{mean}}$) in a section of the spinal cord in images reconstructed from clinical research data using algorithms implemented in the vendor software. To compare against attenuation-corrected data, the spine was manually segmented for each subject and introduced as a bone structure into the pseudo-CTs used for attenuation correction.

## 2 Methods and materials

### 2.1 Simulations

The XCAT mathematical phantom (version 2) (11) was used to generate [${}^{18}$F]FDG tracer distributions of organs in the neck and thorax for a single 25 cm field of view, based on reported uptake in healthy subjects (4, 15–18). We used the XCAT standard male and standard female phantoms. The phantoms were simulated to a voxel size of 2.1×2.1×2.8 mm. Photon attenuation maps of 511 keV were also generated for the region by the XCAT software. These attenuation maps were scaled to units of ${\mathrm{cm}}^{-1}$. Modified attenuation maps were also generated to simulate those derived from Dixon MRI sequences, which was achieved by replacing all bone linear attenuation coefficients $\geq 1.2\;{\mathrm{cm}}^{-1}$ with a muscle linear attenuation coefficient of 0.99 ${\mathrm{cm}}^{-1}$ (19).

Each XCAT distribution was forward-projected using SIRF (20) to generate a sinogram of the distribution. Attenuation correction factors (ACFs) were obtained from the attenuation maps that included bone attenuation coefficients, and scatter was calculated using the single scatter simulation algorithm in STIR (21). The XCAT activity, ACFs, and scatter sinograms were combined to create sinograms simulating acquired PET data (22). Poisson noise was added to the sinogram data by scaling the number of counts in the sinogram to a value representative of the acquired PET data and randomly drawing samples from a Poisson distribution. The sinogram was then scaled back to the original number of counts prior to image reconstruction. Time-of-flight information was not included in simulated data.

ACFs and scatter were also calculated for the attenuation maps without bone for use during image reconstruction. Simulated sinograms were reconstructed using an Ordered Subset Expectation Maximisation (OSEM) algorithm (28 subsets, 2 iterations, voxel size 2×2×2.8 mm) with attenuation and scatter correction. Image reconstruction for each phantom was performed twice: once with attenuation and scatter correction calculated from the attenuation map with bone and a second time with corrections calculated from the attenuation map without bone. PSF modelling was not included.

To perform simulations at scanner resolution, the average distance of the spinal cord to the image centre was measured on patient acquisitions so that NEMA performance results for the scanner could be used to determine an appropriate resolution for our simulation that was representative of spinal cord acquisitions. From an average distance of 2.4 cm, a transaxial resolution of 4.4 mm and an axial resolution of 6 mm, which was simulated by applying a 3D Gaussian filter to the generated XCAT activity distributions and attenuation maps using ImageJ (23). The simulation and image reconstruction process in STIR was repeated as described above for these filtered XCAT phantom distributions to simulate sinogram data and images acquired on a scanner detector resolution representative of a clinical PET/MRI scanner.

#### 2.1.1 Analysis

Five-millimetre ROIs were drawn in the spinal cord at each vertebral level corresponding to C1 to T5 vertebrae. The mean activity and standard deviation were measured for each ROI.

Measurements were averaged for the male and female XCAT phantom images, and a linear regression was performed using the mean measured uptake from each ROI to demonstrate the trend of activity along the length of the spinal cord, with 95% confidence intervals also calculated. A Wilcoxon signed-rank test was used to determine the statistical significance of results, as this analysis is suitable for non-parametric paired data. Results are considered statistically significant when P<0.05.

### 2.2 Clinical acquisition

Imaging was performed on the SIGNA PET/MR scanner (GE HealthCare Inc., Milwaukee, WI, USA) in accordance with the Declaration of Helsinki. The study had ethics committee approval, and all participants gave written informed consent. Two participants, one healthy and one with amyotrophic lateral sclerosis, were administered 250 MBq of [${}^{18}$F]FDG 60 min before acquisition. PET data was acquired at two bed positions for 10 min per bed in head-first supine orientation. MRI was performed simultaneously with PET using the body coil for dedicated attenuation correction [Dixon and ZTE sequences]. The following anatomical sequences were also acquired using a head and neck coil: axial T1-weighted fast spin echo (FSE) and axial T2-weighted fluid attenuated inversion recovery (FLAIR) for the brain and sagittal T2-weighted FSE, sagittal T1-weighted FLAIR, and axial T2-weighted FSE of the spinal cord.

PET image reconstruction was performed offline using the vendor-provided software Duetto version 02.18; the reconstruction process used both OSEM and time-of-flight OSEM (TOFOSEM) algorithms with 28 subsets and 2 iterations, resulting in a voxel size of 2.3×2.3×2.8 mm. Reconstructions with PSF modelling were performed separately using the same parameters. MR-derived attenuation correction was used with ZTE sequences for the head and built-in templates for MR coils (24). Images were reconstructed under two conditions: without any post-filtering and with a 5-mm transaxial Gaussian filter and three-point axial convolution filter of [1 4 1] applied post-reconstruction. All parameters were chosen to be representative of clinical use cases.

Reconstructions were also performed using the Q.Clear algorithm (Equation 1), a Bayesian penalised likelihood reconstruction method (GE HealthCare Inc.) with a single, user-modifiable parameter, beta (b). Q.Clear and TOF Q.Clear were performed with b values of 100, 200, and 400, all of which were initialised using a two-iteration OSEM reconstruction. This dictates the strength of the prior as shown in the objective function when using a Bayesian a priori algorithm such as Q-Clear:

(1) $$\hat{\lambda }={\arg\;\max}_{\lambda \geq 0}\sum_{i=1}^{n_{d}}\{y_{i}\log[a\lambda {]}_{i}-[a\lambda {]}_{i}-\beta R(\lambda )\}$$

where *λ* corresponds to the image voxel valye, *α* to the system matrix, *R*(*λ*) is the prior and *b* is the weighting applied to the prior. The statistical prior imposes expected properties that the resulting image should adhere to, and in this case, it allows for smoothing over a local voxel neighbourhood to reduce image noise. While Q.Clear reconstruction improves the signal-to-noise ratio and visual detection of lesions, previous work to quantify measured activity and contrast recovery has shown that b≤400 is required for features witha diameter of less than 10 mm without losing signal intensity and the contrast-to-noise ratio (25). Therefore, optimisation of higher beta values was not pursued in this study.

#### 2.2.1 Introducing vertebral bone to attenuation correction

Another avenue of investigation into the effects of vertebral bone in attenuation correction involved introducing vertebral bone into the image reconstruction process for participant acquisitions. The cortical bone in the vertebra was manually segmented using 3DSlicer 5.6.1 software (26); this segmentation was performed on ZTE and anatomical T1 and T2 images acquired simultaneously with the PET scan for study participants.

Pseudo-CTs derived from Dixon MR images, from which attenuation maps are calculated and are displayed in Hounsfield units (HU), were modified by introducing the vertebral bone segmentations as areas with 800 HU (27). These modified pseudo-CTs were then used for attenuation and scatter correction, and image reconstruction was performed as described in Section 2.2.

#### 2.2.2 Analysis

Activity is normalised to body weight and displayed as standardised uptake values (${\mathrm{SUV}}_{\mathrm{bw}}$), which are used for all results presented in this part of the study. Regions of interest (ROIs), 5 mm in diameter, were drawn in the spinal cord at each vertebral level from C1 to T4 on T2-weighted MRI images and used to measure the mean SUV (${\mathrm{SUV}}_{\mathrm{mean}}$) and standard deviation for each ROI in the PET images.

The ${\mathrm{SUV}}_{\mathrm{mean}}$ was averaged over patient acquisitions, and a linear regression was performed to demonstrate the trend of activity along the length of the spinal cord. A Wilcoxon signed-rank test was used to determine the statistical significance of results, with Bonferroni corrections applied where tests were applied to compare several different results. The Bonferroni correction is a conservative safeguard against type 1 errors in statistical analysis, and it works by dividing the usually accepted significance value of P>0.05 by the number of times the test is run.

## 3 Results

### 3.1 XCAT simulations

**Figure 1:**
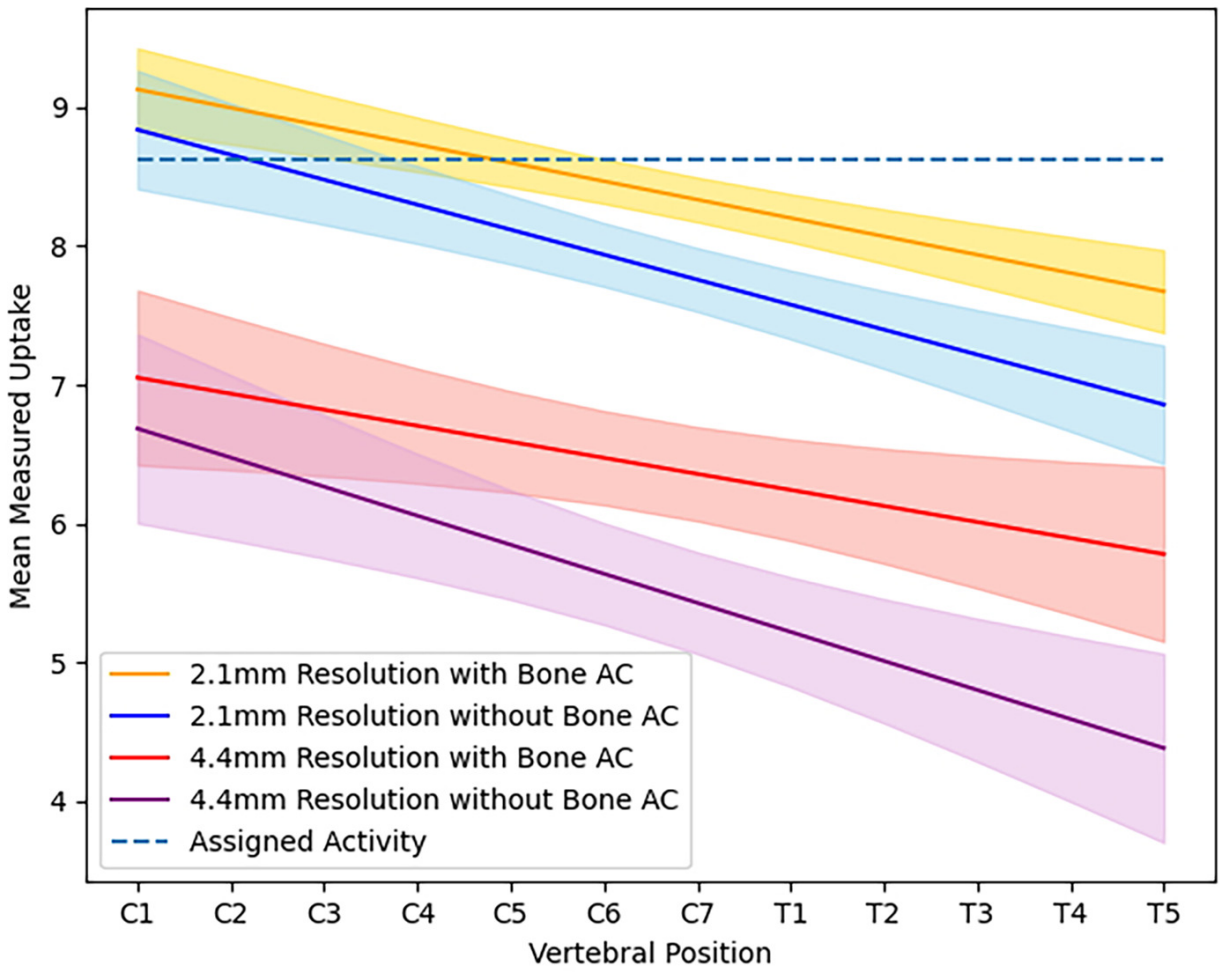
Average activity measured in the spinal cord at each vertebral position for images of simulated XCAT phantoms at high resolution (2×2×2.8 mm) and scanner resolution (4.2×4.2×6.1 mm) when reconstructed using an attenuation map with or without bone.

For the XCAT phantom simulated at 2 mm, a decreasing pattern of uptake was measured along the length of the spinal cord, as shown in Figure 1, despite a constant value initially being assigned in the XCAT distribution. When bone features were maintained in attenuation and scatter correction, measured uptake in the cervical spine was more consistent with the initial assigned value, but it still decreased in the thoracic spinal cord. For images reconstructed without bone included in attenuation maps, the measured uptake was overall lower then the fully attenuation-corrected images, with the decrease ranging from 3% to 6.7% in the cervical spinal cord and 9.5% to 10.4% in the thoracic spinal cord (P<0.001).

**Figure 2:**
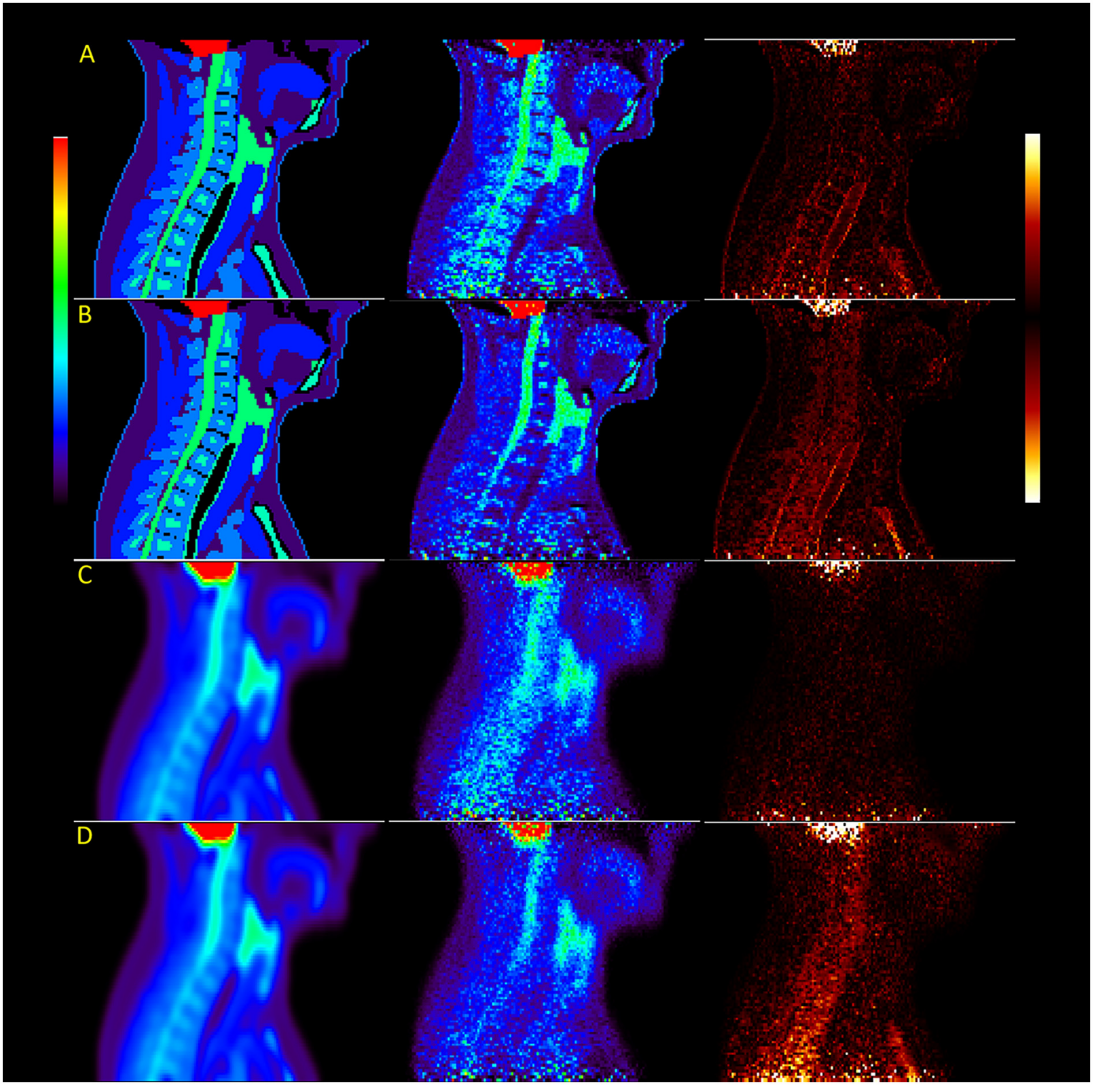
Images of the XCAT male phantom reconstructed with OSEM at high resolution (rows **A** and **B**) and scanner resolution (rows **C** and **D**), with rows **B** and **D** using attenuation maps without bone. The last image in each row shows the difference between reconstruction and original simulated distributions.

Images reconstructed from sinograms of the XCAT phantom simulated with a 4.2 mm detector resolution show a more rapidly decreasing trend in measured uptake along the length of the spinal cord compared to the higher-resolution images, also shown visually in Figure 2, where the difference between the original distribution and reconstructed images is higher in the thoracic spine. When comparing different simulated resolutions, this is measured at a difference of 17.8%–32.2% for fully attenuation and scatter-corrected images (P<0.001). The difference in measured activity in the spinal cord between the attenuation correction methods is also greater, giving a 6.4%–14.8% difference in the cervical spine and 19.4%–23.9% difference in the thoracic spine (P<0.001).

### 3.2 Clinical acquisitions

**Figure 3:**
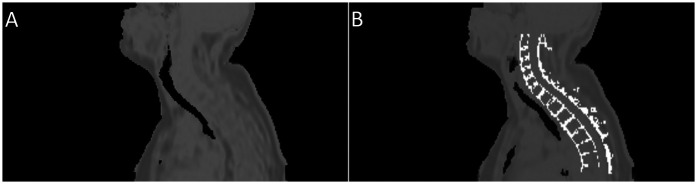
Example of the pseudo-CTs used to derive attenuation maps. Image **(A)** shows the pseudo-CT created from a Dixon MRI sequence by the vendor software, while image **(B)** shows the same psuedo-CT with segmented cortical bone from the spine added.

**Figure 4:**
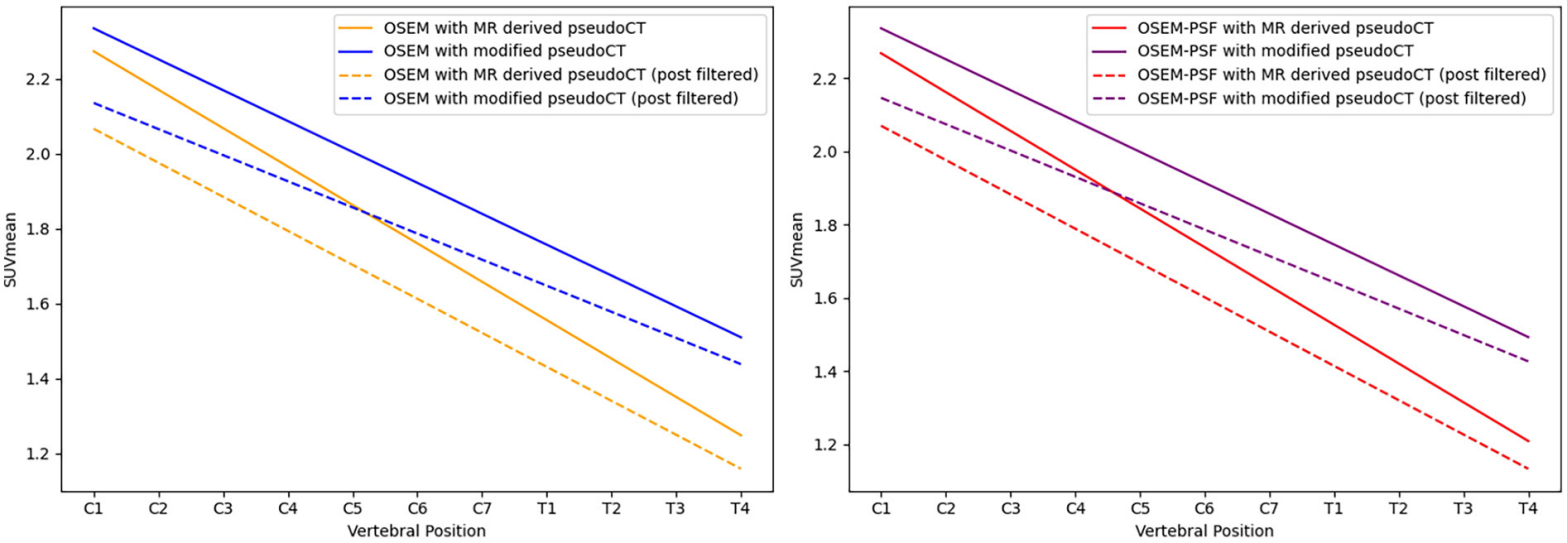
Linear regression of the ${\mathrm{SUV}}_{\mathrm{mean}}$ along the length of the spinal cord for images reconstructed using OSEM (left) and OSEM incorporating PSF modelling (right), both showing measurements for images with and without the vertebral bone segmentation included in the attenuation map. Dashed lines indicate measurements obtained from post-filtered images.

Figure 3 provides an example of the modified pseudo-CT, which incorporates vertebral bone segmented from MR images. The ${\mathrm{SUV}}_{\mathrm{mean}}$ in images reconstructed with vertebral bone added to pseudo-CTs for attenuation correction showed an increase across all algorithms compared to using the original MR-derived pseudo-CT. In non-TOF OSEM reconstructions, this corresponded to a 1.7%–11% increase in the ${\mathrm{SUV}}_{\mathrm{mean}}$ in the cervical spinal cord and 10.7%–16.4% in the thoracic spinal cord (P<0.001) when the spine was included for attenuation correction, as demonstrated by the trends displayed in Figure 4.

**Figure 5:**
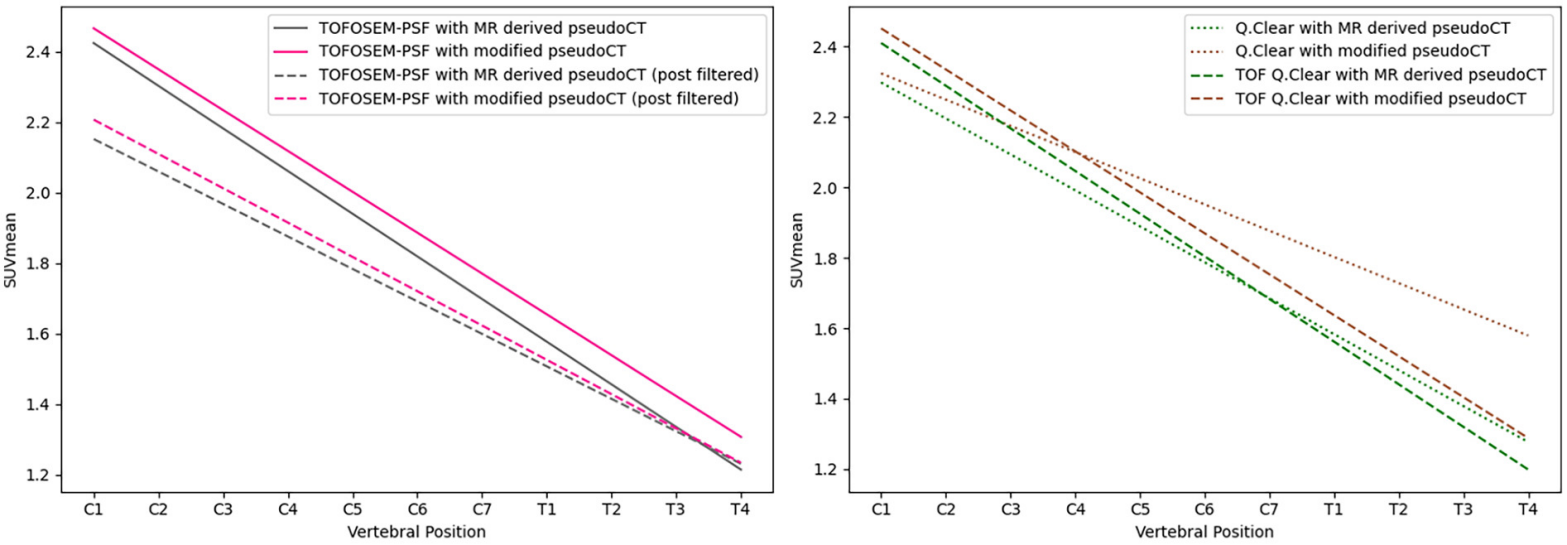
Linear regression of the ${\mathrm{SUV}}_{\mathrm{mean}}$ along the length of the spinal cord for images reconstructed with TOFOSEM (left) and TOFOSEM including PSF modelling (right), both showing measurements for images with and without the vertebral bone segmentation included in the attenuation map. Dashed lines in the TOFOSEM-PSF plot indicate measurements obtained from post-filtered images.

**Figure 6:**
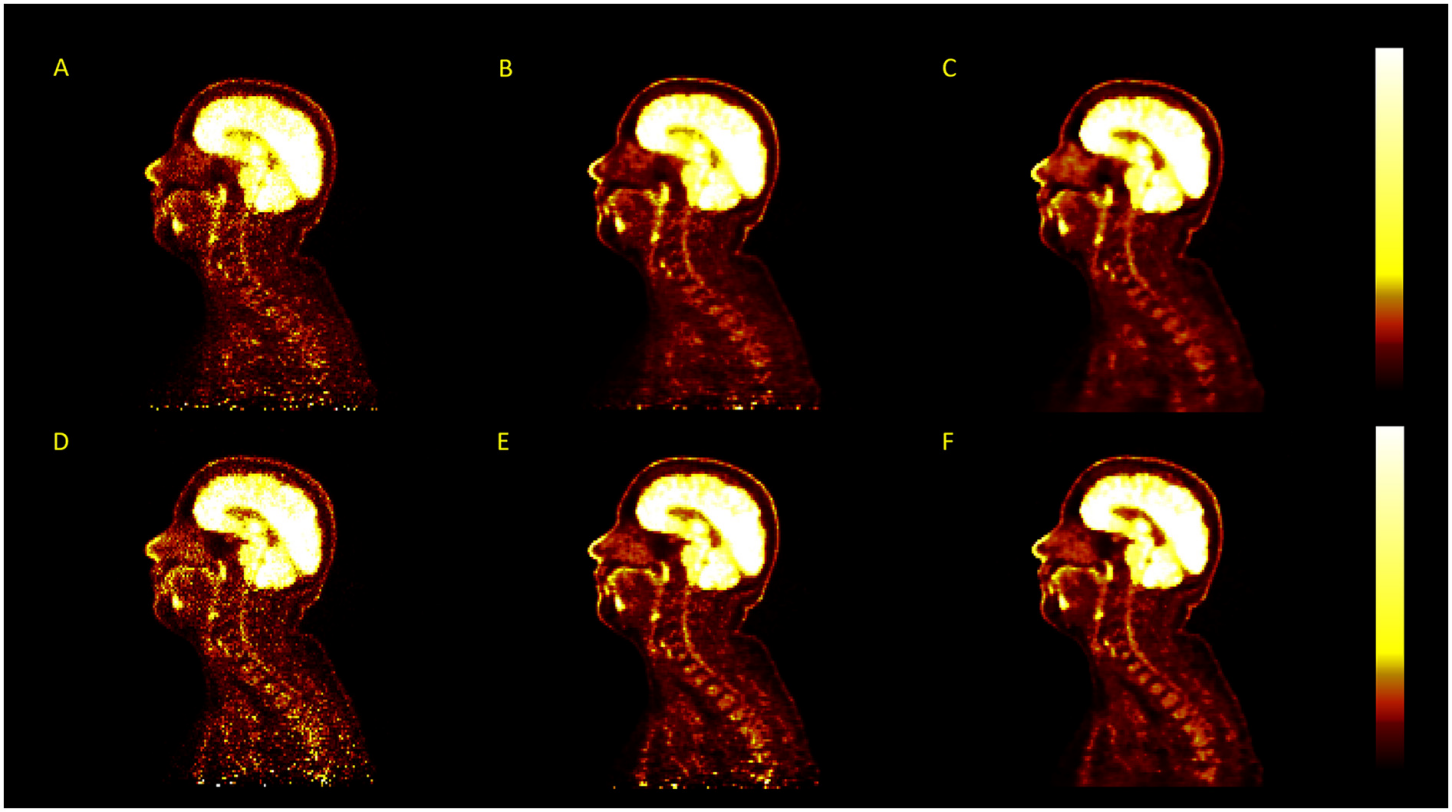
Examples of PET images reconstructed using different image reconstruction algorithms for the same acquisition, with no post-filtering applied, displayed in ${\mathrm{SUV}}_{\mathrm{bw}}$. The algorithms used are OSEM **(A)**, OSEM-PSF **(B)**, Q.Clear **(C)**, TOFOSEM **(D)**, TOFOSEM-PSF **(E)**, and TOF Q.Clear **(F)**.

Using TOF algorithms, as shown in Figure 5, reduced the difference between reconstructions with and without vertebral bone attenuation to 0.7%–6.6% for TOFOSEM-PSF (P<0.001) compared to the 1.9%–17.2% difference when OSEM-PSF is used. Changes to the ${\mathrm{SUV}}_{\mathrm{mean}}$ in the spinal cord following TOF correction were not considered significant compared to the same algorithm without TOF (0.03≤P≤0.7 for compared algorithms, with significance taken to be α=0.017 following Bonferroni correction for three comparisons). This is highlighted in Figure 6, which displays images from the same patient and same pseudo-CT reconstructed with each available algorithm, showing no obvious differences in the spinal cord.

**Figure 7:**
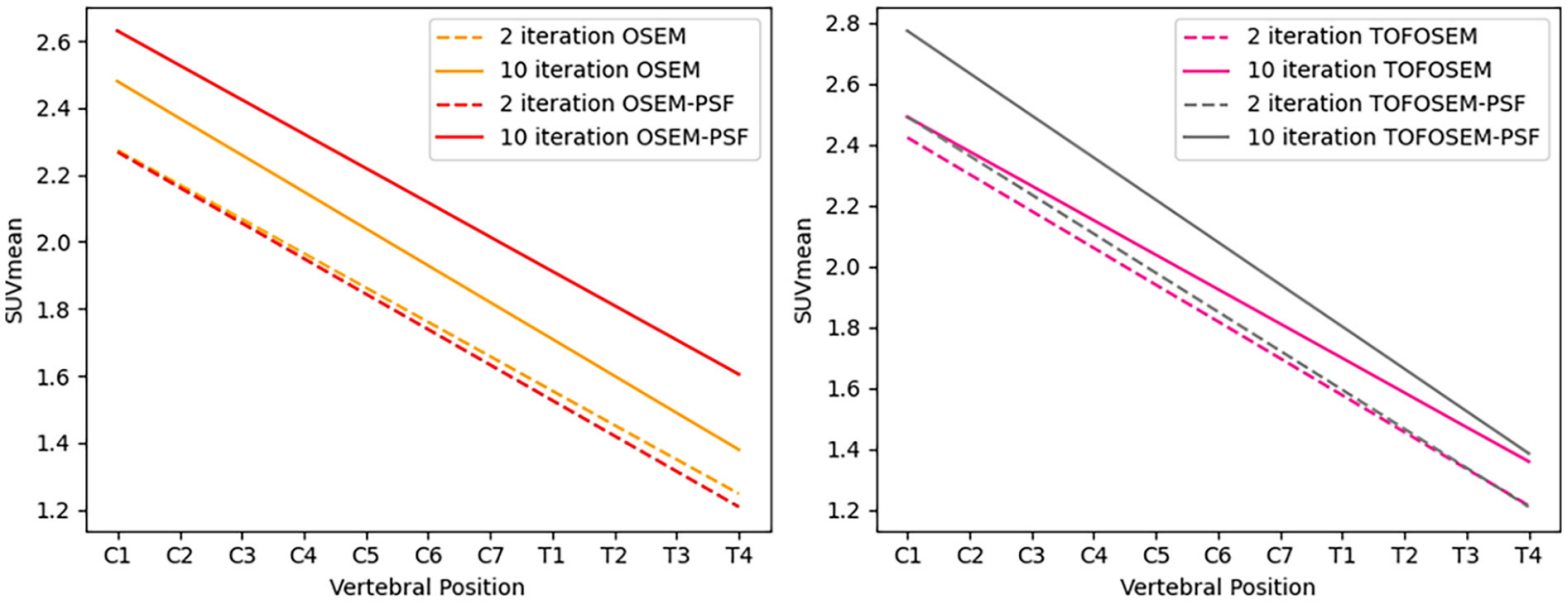
Linear regression of the ${\mathrm{SUV}}_{\mathrm{mean}}$ along the length of the spinal cord for images reconstructed with OSEM (left) and TOFOSEM (right) with and without PSF, both showing measurements for images with and without the vertebral bone segmentation included in the attenuation map. Dashed lines indicate measurements for images reconstructed to 2 iterations, while the solid line shows the same measurements in images reconstructed to 10 iterations.

**Figure 8:**
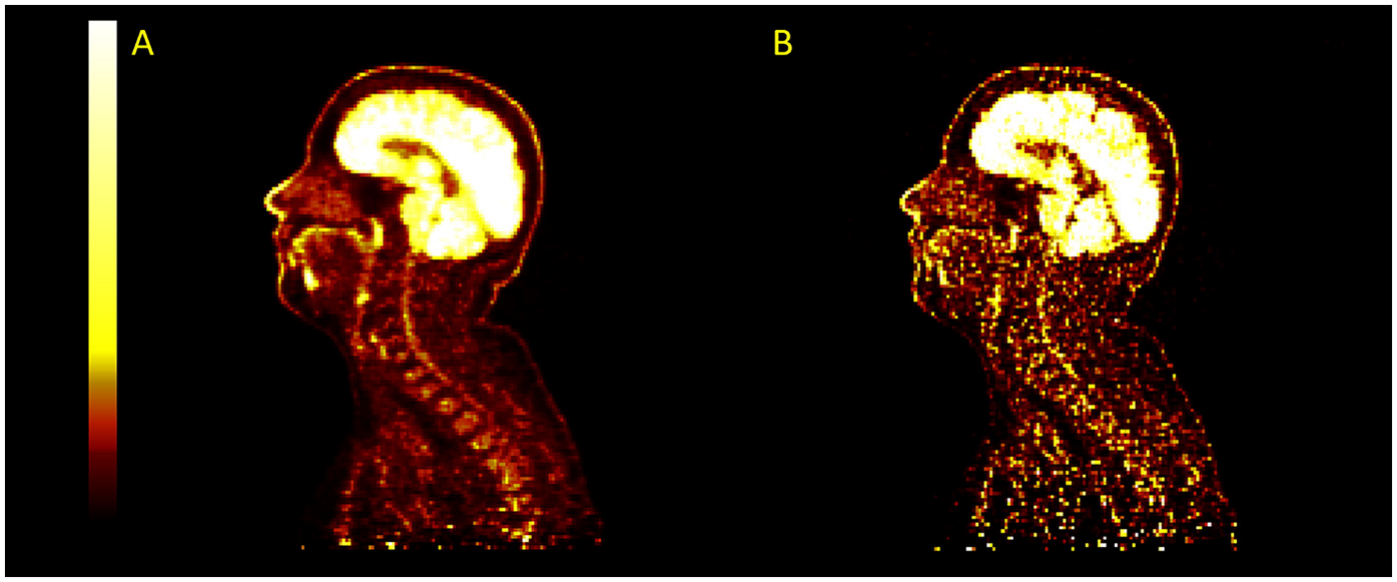
Example images for images reconstructed with TOFOSEM-PSF at 2 iterations **(A)** and 10 iterations **(B)**. The image becomes sharper and recover more activity in the spinal cord at higher iterations but with a large increase in image noise.

Applying PSF corrections does not introduce any change to the trend in activity along the length of the spinal cord (P=0.6 for MR-derived pseudo-CT and P=0.9 for modified pseudo-CT) or impact the comparison between attenuation correction approaches at the chosen paramters of 2 iterations and 28 subsets. However, as shown in Figure 7, an increase in the number of iteration images reconstructed with PSF correction did lead to an increase in measured activity for OSEM and TOF-OSEM algorithms (P=0.01 and P=0.05, respectively, at 10 iterations). However, as displayed in Figure 8, increasing the number of iterations significantly increases noise in the final images.

**Figure 9:**
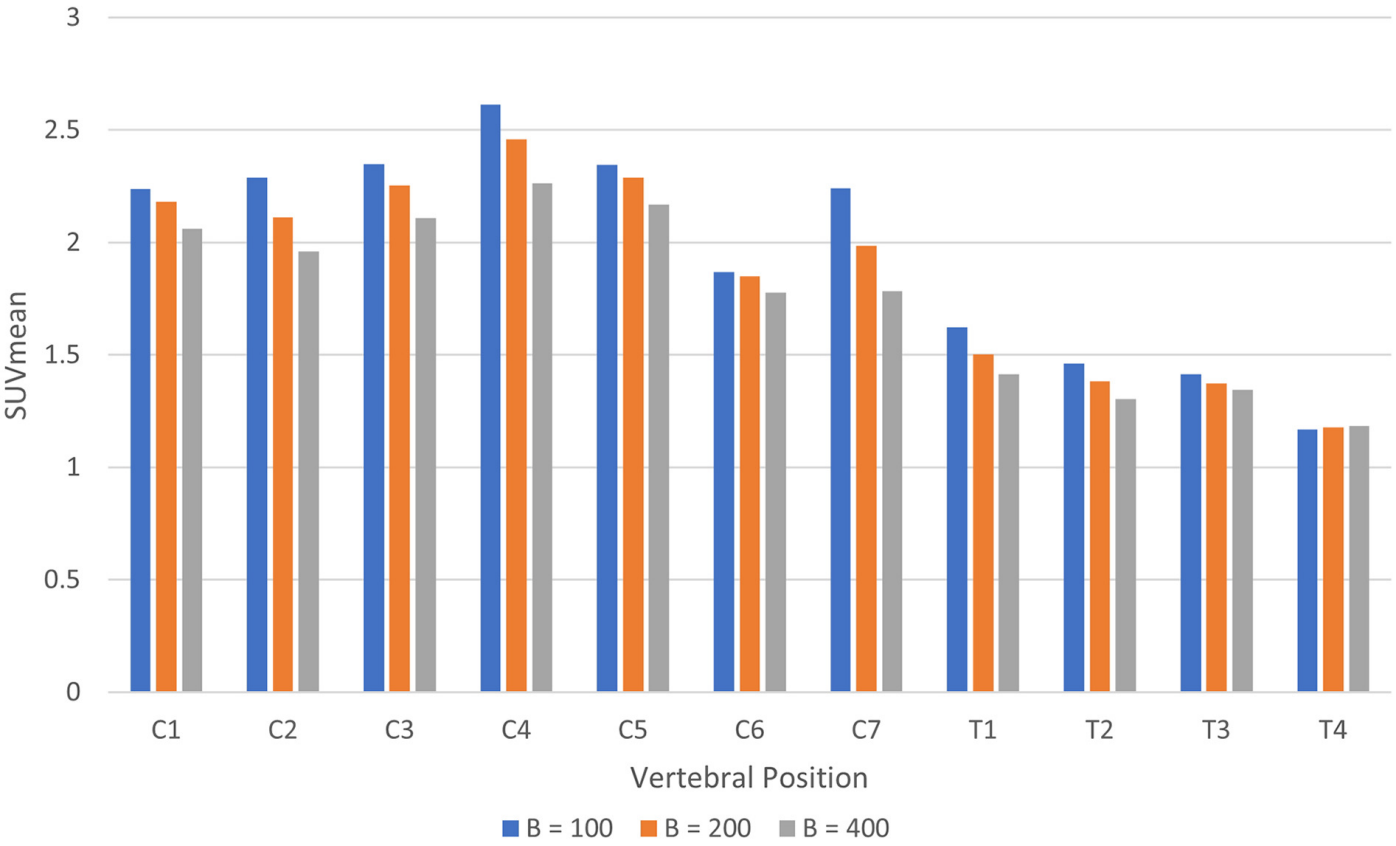
Histogram of ${\mathrm{SUV}}_{\mathrm{mean}}$ in the spinal cord by vertebral position for images reconstructed using TOF Q.Clear with beta = 100, 200, and 400.

The same trends were observed in images reconstructed using the Q.Clear algorithm, as demonstrated in Figure 5. Changing beta in TOF Q.Clear reconstructions introduced small quantitative changes (P<0.001 for β=100 to β=400 and P=0.002 for all other comparisons), with the ${\mathrm{SUV}}_{\mathrm{mean}}$ decreasing with increasing beta, as shown in Figure 9. Among the beta values tested, assigning β=100 yielded the smallest difference in the ${\mathrm{SUV}}_{\mathrm{mean}}$ between images reconstructed with the MR-derived and modified pseudo-CTs for attenuation correction, ranging from 0.6% to 5.2%. This result was the smallest range of difference in ${\mathrm{SUV}}_{\mathrm{mean}}$ found in this study when comparing images reconstructed with different attenuation correction pseudo-CTs.

**Figure 10:**
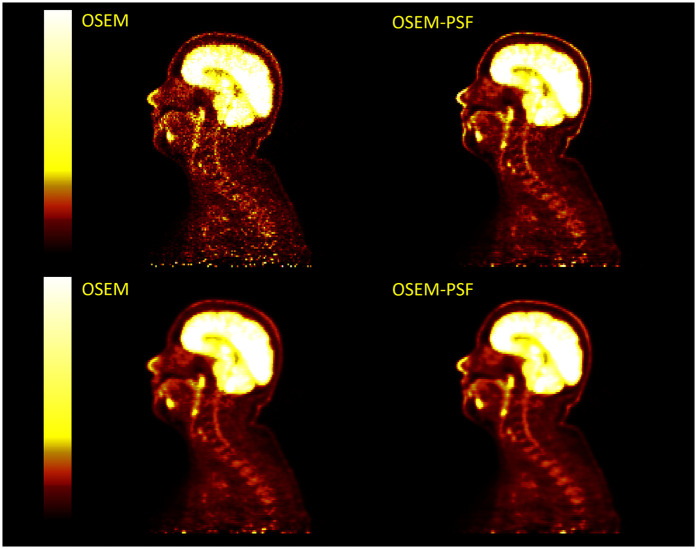
Four PET images in two columns compare OSEM and OSEM-PSF techniques without any filtering (top row) and with post-filtering (bottom row). Each image shows a side view scan of the same head and neck with a vertical color scale ranging from dark red to bright yellow, indicating intensity levels.

**Figure 11:**
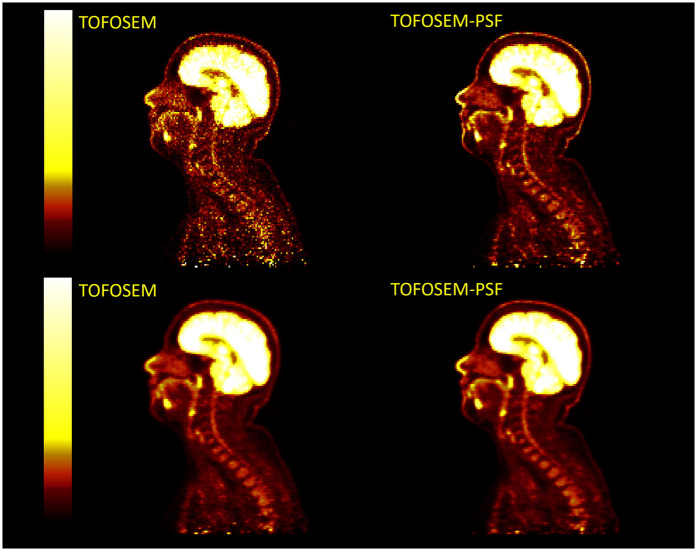
Examples of PET images with post-filtering (5 mm transaxial Gaussian filter and 3 point axial convolution [1 4 1] filter) applied (bottom row) to TOFOSEM reconstructed images with and without PSF modelling, displayed in ${\mathrm{SUV}}_{\mathrm{bw}}$.

Example images for each OSEM algorithm, with and without post-filtering, are displayed in Figures 10 and 11. Images with post-filtering applied demonstrate the same trends in the ${\mathrm{SUV}}_{\mathrm{mean}}$ along the spinal cord but with a reduction in measured activity of 1.1%–20.7%. This reduction was only considered significant for reconstructions that used PSF modelling (P<0.001 OSEM-PSF, P=0.002 for TOFOSEM-PSF), as shown in Figures 4 and 5. The differences between images reconstructed with and without vertebral bone in the attenuation maps were also consistent with the unfiltered images. In time-of-flight reconstructions, the difference in ${\mathrm{SUV}}_{\mathrm{mean}}$ between spinal cord ROIs in equivalent images when post-filtering was applied was greater, up to 20.7% of TOFOSEM and 17.9% for TOFOSEM-PSF, than the difference measured when applying different attenuation correction approaches (range 0.7%–6.6%).

## 4 Discussion

XCAT simulation results indicate that disregarding bone in attenuation correction maps affects the quantification of the activity in the spinal cord, with a statistically significant decrease of up to 10.4% in measured uptake when compared to fully attenuation-corrected images. This is in line with a previous study (28) reporting on the impact of MR-derived attenuation correction on SUV in spine lesions, concluding that the impact of removing bone from attenuation maps depends on the proximity of the region of interest to bone. Detector resolution also plays a crucial role in this, reducing overall measured activity by up to 32.2% relative to the true uptake, which we conclude is due to the partial volume effect. This effect acts in combination with the choice of attenuation correction, leading to a further reduction in activity when bone is ignored in MR-derived attenuation maps, particularly in the thoracic spine. This consideration is important because previous literature has reported a decreased pattern of uptake along the length of the spinal cord as a physiological phenomenon (3, 4). While other evidence suggests that this may be true for [${}^{18}$F]FDG uptake (2, 3), research is ongoing into novel central nervous system tracers for which this is not the case (29). Therefore, the effects presented in this study may confound results for these studies.

Time of flight was not considered as part of the simulation phase of this study, which would also improve the localisation of detected photons. These results suggest that partial volume effects do occur in spinal cord PET, as previously proposed (2), and that partial volume correction should also improve quantification in the spinal cord. One way in which this is commonly performed is by detector response modelling, which was not considered during our simulation due to the lack of an appropriate model in the current implementation of SIRF.

In patient data, the addition of cortical bone from the vertebra to attenuation maps led to an overall increase in measured uptake in the spinal cord of up to 17.2%. Although this difference was not as large as demonstrated in simulated data, it is in line with previous work investigating five-tissue class attenuation maps for spinal cord PET/MR (30). OSEM and OSEM-PSF reconstructions exhibited the largest disparity between images reconstructed with or without vertebral bone. Inserting segmented bone features into attenuation maps can be prone to errors when applying a Hounsfield unit that is not patient-specific (31). MR-derived segmentation can also be affected by patient motion (32), a factor that is not accounted for in this study. However, in the absence of same-patient CT acquisitions, this approach provides a useful example of the role vertebral bone plays in attenuation correction. The PSF modelling used in this software is based on the scanner model and includes spatial variation across the field of view, but introducing PSF modelling did not make a significant difference to measured activity in the spinal cord for the reconstruction parameters used. The OSEM parameters were chosen to reflect common clinical practice; however, it has been shown that applying PSF in EM algorithms can slow convergence in early iterations (33). Subsequently, we demonstrated that applying PSF corrections when reconstructing PET data does lead to an increase in ${\mathrm{SUV}}_{\mathrm{mean}}$ in images reconstructed with OSEM and TOFOSEM to 10 iterations; however, this must be balanced with increased image noise and computation time. The reason why PSF modelling appears to be insufficient in recovering the signal remains unclear.

TOF correction reduced the impact of including vertebral bone in attenuation correction on measured uptake, suggesting that time of flight in PET-MR can overcome the barriers posed by the lack of CT or transmission-based attenuation correction. This finding aligns with previously reported results in other organs (12). Applying post-filtering reduced measured uptake in all cases, with a maximum reduction of 20.7%. In TOF reconstructions, this had a greater impact on ${\mathrm{SUV}}_{\mathrm{mean}}$ in the spinal cord than changing the attenuation correction approach, which showed a maximum change of 6.6% in ${\mathrm{SUV}}_{\mathrm{mean}}$. For small-diameter structures, care should be taken when performing quantification on post-filtered images. This could be due to improved contrast recovery and faster convergence in TOF reconstructions, as emission data is better localised (12, 13). Since PET image reconstruction is a complex problem with multiple sources of error, including those arising from attenuation and scatter, using TOF algorithms may also lead to the effects of these errors becoming more localised. A further assessment against a known ground truth image would help in determining the impact that TOF algorithms have on quantification across the whole image.

The Q.Clear algorithm results displayed the same trends as OSEM, with TOF reducing the difference in ${\mathrm{SUV}}_{\mathrm{mean}}$ between different attenuation correction maps from a maximum of 18.3% with Q.Clear to a maximum of 6.2% with TOF Q.Clear at beta = 200. Increasing the beta value decreased the measured uptake in the spinal cord, in agreement with previously reported results (25, 34). The images reconstructed with beta = 100 gave the lowest difference between the two attenuation maps, which also corresponds to the previous studies suggesting that a beta value of 100–200 is more appropriate for applications involving small (<10 mm) regions of interest when using Q.Clear. Therefore, Q.Clear could be optimised for spinal cord reconstruction by using low values of beta to avoid over-smoothing, given the small diameter of the spinal cord.

Accurate quantitative spinal cord PET/MRI would be beneficial for translational neuroscience research by providing complimentary functional information that is acquired simultaneously, particularly with the increasing number of specific PET tracers for imaging the central nervous system (29, 35) and myelin-binding (36) radiopharmaceuticals. This could improve the understanding of disease mechanisms and help assess the therapeutic efficacy of treatments in clinical trials by allowing for monitoring of receptor activity and neuronal structure in the spinal cord (37). This article has focused on investigating the sources of error in PET data to facilitate a move towards more accurate quantification of spinal cord PET in PET/MRI systems.

### 4.1 Future work

Future work in this area should focus on investigating resolution recovery and partial volume correction that can be applied to PET data in PET/MRI. Hybrid reconstruction algorithms, which use anatomical MRI as a prior for PET data reconstruction (38), could be beneficial in maintaining the distinction between the spinal cord and surrounding active tissue in PET/MRI and has previously been implemented for time-of-flight PET data (39).

Numerous partial volume correction and resolution recovery methods using features from other imaging modalities have also been published (40, 41), with specific interest in this area for application with PET/MR scanners, allowing for readily available spatially registered MR images acquired simultaneously with PET data (42). Further investigations into applying these to spinal cord PET could prove advantageous, given the high resolution and tissue contrast available in the spine through MRI (43).

## 5 Conclusion

In this study, we demonstrated that in systems without time of flight, ignoring vertebral bone in PET/MRI leads to an underestimation of tracer uptake in the spinal cord by up to 23.9%, particularly in the thoracic spine. We also demonstrated that for a system with a 4.4 mm resolution, measured PET uptake in the spinal cord is reduced by up to 32.2% compared to higher-resolution systems. We conclude that this is due to partial volume effects, which have greater impact on quantification than ignoring bone in attenuation correction. Applying TOF correction can reduce disparity in ${\mathrm{SUV}}_{\mathrm{mean}}$ between images reconstructed with and without vertebral bone in attenuation maps to a range of 0.7%–6.6%. Applying PSF modelling to both OSEM and TOFOSEM reconstruction methods requires a higher number of iterations than is often used in clinical practice to recover measured uptake; however, reconstruction with the Q.Clear algorithm was effective at recovering activity. More research is needed on the best way to apply partial volume correction in PET imaging to facilitate accurate quantification of PET tracer uptake in the spinal cord in PET/MRI.

## Data Availability

The datasets presented in this article are not readily available because datasets are available by request to the first author. Requests to access the datasets should be directed to Steven Sourbron, s.sourbron@sheffield.ac.uk.

## References

[1] Blasco H Vourch P Pradat PF Gordon PH Andres CR Corcia P. Further development of biomarkers in amyotrophic lateral sclerosis. Expert Rev Mol Diagn. (2016) 16(8):853–68. 10.1080/14737159.2016.1199277 27275785

[2] Kiamanesh Z Banezhad F Nasiri Z Emami F Treglia G Sadeghi R. Physiological distribution of 18F-FDG in the spinal cord: a systematic review. J Spinal Cord Med. (2021) 44(4):517–24. 10.1080/10790268.2019.1672954 31682787 PMC8288118

[3] Do BH Mari C Tseng JR Quon A Rosenberg J Biswal S. Pattern of 18F-FDG uptake in the spinal cord in patients with non-central nervous system malignancy. Spine. (2011) 36(21):E1395–401. 10.1097/BRS.0B013E31820A7DF8 21311407

[4] Patel NJ Gupta V Vibhute PG Jain MK Accurso JM. A large cohort study of 18F fluoro-deoxy-glucose uptake in normal spinal cord: quantitative assessment of the contamination from adjacent vertebral marrow uptake and validity of normalizing the cord uptake. J Comput Assist Tomogr. (2017) 41(1):125–30. 10.1097/RCT.0000000000000479 27560019

[5] Aiello M Alfano V Salvatore E Cavaliere C Picardi M Della Pepa R [18F]FDG uptake of the normal spinal cord in PET/MR imaging: comparison with PET/CT imaging. EJNMMI Res. (2020) 10(1):91. 10.1186/s13550-020-00680-8 32761394 PMC7410944

[6] Catana C Van Der Kouwe A Benner T Michel CJ Hamm M Fenchel M Toward implementing an MRI-based PET attenuation-correction method for neurologic studies on the MR-PET brain prototype. J Nucl Med. (2010) 51(9):1431–8. 10.2967/JNUMED.109.069112 20810759 PMC3801218

[7] Sgard B Khalifé M Bouchut A Fernandez B Soret M Giron A ZTE MR-based attenuation correction in brain FDG-PET/MR: performance in patients with cognitive impairment. Eur Radiol. (2020) 30(3):1770–9. 10.1007/s00330-019-06514-z 31748857

[8] Prakken NHJ Besson FL Borra RJH Büther F Buechel RR Catana C PET/MRI in practice: a clinical centre survey endorsed by the European association of nuclear medicine (EANM) and the EANM forschungs GmbH (EARL). Eur J Nucl Med Mol Imaging. (2023) 50(10):2927–34. 10.1007/S00259-023-06308-Y/TABLES/2 37378857

[9] Galve P Rodriguez-Vila B Herraiz J García-Vázquez V Malpica N Udias J Recent advances in combined positron emission tomography and magnetic resonance imaging. J Instrum. (2024) 19(01):C01001. 10.1088/1748-0221/19/01/C01001

[10] Karakatsanis NA Abgral R Trivieri MG Dweck MR Robson PM Calcagno C Hybrid PET- and MR-driven attenuation correction for enhanced 18F-NaF and 18F-FDG quantification in cardiovascular PET/MR imaging. J Nucl Cardiol. (2020) 27(4):1126–41. 10.1007/S12350-019-01928-0/FIGURES/9 31667675 PMC7190435

[11] Segars WP Sturgeon G Mendonca S Grimes J Tsui BMW. 4D XCAT phantom for multimodality imaging research. Med Phys. (2010) 37(9):4902–15. 10.1118/1.3480985 20964209 PMC2941518

[12] Mehranian A Zaidi H. Impact of time-of-flight PET on quantification errors in MR imaging-based attenuation correction. J Nucl Med. (2015) 56(4):635–41. 10.2967/JNUMED.114.148817 25745090

[13] Lois C Jakoby BW Long MJ Hubner KF Barker DW Casey ME An assessment of the impact of incorporating time-of-flight information into clinical PET/CT imaging. J Nucl Med. (2010) 51(2):237–45. 10.2967/jnumed.109.068098 20080882 PMC2818518

[14] Schramm G. Reconstruction-free positron emission imaging: fact or fiction? Front Nucl Med. (2022) 2:936091. 10.3389/fnume.2022.936091 39354988 PMC11440944

[15] Shen S Wang H Xue Y Yuan L Zhou X Zhao Z Freeform fabrication of tissue-simulating phantom for potential use of surgical planning in conjoined twins separation surgery. Sci Rep. (2017) 7(1):11048. 10.1038/s41598-017-08579-6 28887492 PMC5591222

[16] Zincirkeser S Şahin E Halac M Sager S. Standardized uptake values of normal organs on 18F-fluorodeoxyglucose positron emission tomography and computed tomography imaging. J Int Med Res. (2007) 35(2):231–6. 10.1177/147323000703500207 17542410

[17] Ramos CD Erdi YE Gonen M Riedel E Yeung HWD Macapinlac HA FDG-PET standardized uptake values in normal anatomical structures using iterative reconstruction segmented attenuation correction and filtered back-projection. Eur J Nucl Med. (2001) 28(2):155–64. 10.1007/S002590000421/METRICS 11303885

[18] Heusch P Buchbender C Beiderwellen K Nensa F Hartung-Knemeyer V Lauenstein TC Standardized uptake values for [18F] FDG in normal organ tissues: comparison of whole-body PET/CT and PET/MRI. Eur J Radiol. (2013) 82(5):870–6. 10.1016/J.EJRAD.2013.01.008 23394765

[19] Dendy P Heaton B. Physics of Diagnostic Radiology. 3rd ed. Bristol, UK: IOP Publishing (2012).

[20] Ovtchinnikov E Brown R Kolbitsch C Pasca E da Costa-Luis C Gillman AG SIRF: synergistic image reconstruction framework. Comput Phys Commun. (2020) 249:107087. 10.1016/J.CPC.2019.107087

[21] Thielemans K Tsoumpas C Mustafovic S Beisel T Aguiar P Dikaios N STIR: software for tomographic image reconstruction release 2. Phys Med Biol. (2012) 57(4):867–83. 10.1088/0031-9155/57/4/867 22290410

[22] Tsoumpas C Buerger C King AP Mollet P Keereman V Vandenberghe S Fast generation of 4D PET-MR data from real dynamic MR acquisitions. Phys Med Biol. (2011) 56(20):6597–613. 10.1088/0031-9155/56/20/005 21937775

[23] Schneider CA Rasband WS Eliceiri KW. NIH image to imageJ: 25 years of image analysis. Nat Methods. (2012) 9(7):671–5. 10.1038/nmeth.2089 22930834 PMC5554542

[24] Eldib M Oesingmann N Faul DD Kostakoglu L Knešaurek K Fayad ZA. Optimization of yttrium-90 PET for simultaneous PET/MR imaging: a phantom study. Med Phys. (2016) 43(8Part1):4768–74. 10.1118/1.4958958 27487894

[25] Lysvik EK Mikalsen LTG Rootwelt-Revheim ME Emblem KE Hjørnevik T. Optimization of Q.Clear reconstruction for dynamic 18F PET imaging. EJNMMI Phys. (2023) 10(1):1–13. 10.1186/S40658-023-00584-1/TABLES/1 37861929 PMC10589167

[26] Fedorov A Beichel R Kalpathy-Cramer J Finet J Fillion-Robin JC Pujol S 3D Slicer as an image computing platform for the quantitative imaging network. Magn Reson Imaging. (2012) 30(9):1323–41. 10.1016/J.MRI.2012.05.001 22770690 PMC3466397

[27] Ceh J Youd T Mastrovich Z Peterson C Khan S Sasser TA Bismuth infusion of ABS enables additive manufacturing of complex radiological phantoms and shielding equipment. Sensors. (2017) 17(3):459. 10.3390/s17030459 28245589 PMC5375745

[28] Samarin A Burger C Wollenweber SD Crook DW Burger IA Schmid DT PET/MR imaging of bone lesions – implications for PET quantification from imperfect attenuation correction. Eur J Nucl Med Mol Imaging. (2012) 39(7):1154–60. 10.1007/S00259-012-2113-0/FIGURES/5 22526955

[29] Arlicot N Vercouillie J Ribeiro MJ Tauber C Venel Y Baulieu JL Initial evaluation in healthy humans of [18F]DPA-714, a potential PET biomarker for neuroinflammation. Nucl Med Biol. (2012) 39(4):570–8. 10.1016/J.NUCMEDBIO.2011.10.012 22172392

[30] Brancato V Borrelli P Alfano V Picardi M Mascalchi M Nicolai E The impact of MR-based attenuation correction in spinal cord FDG-PET/MR imaging for neurological studies. Med Phys. (2021) 48:5924–34. 10.1002/MP.15149 34369590 PMC9293017

[31] Schleyer PJ Schaeffter T Marsden PK. The effect of inaccurate bone attenuation coefficient and segmentation on reconstructed PET images. Nucl Med Commun. (2010) 31(8):708–16. 10.1097/MNM.0B013E32833B0573 20505553

[32] Buerger C Tsoumpas C Aitken A King AP Schleyer P Schulz V Investigation of MR-based attenuation correction and motion compensation for hybrid PET/MR. IEEE Trans Nucl Sci. (2012) 59(5):1967–76. 10.1109/TNS.2012.2209127

[33] Thielemans K Asma E Ahn S Manjeshwar RM Deller T Ross SG Impact of PSF modelling on the convergence rate and edge behaviour of EM images in PET. In: *IEEE Nuclear Science Symposium Conference Record*. (2010). p. 3267–72. 10.1109/NSSMIC.2010.5874409

[34] Reynés-Llompart G Gámez-Cenzano C Vercher-Conejero JL Sabaté-Llobera A Calvo-Malvar N Martí-Climent JM. Phantom, clinical, and texture indices evaluation and optimization of a penalized-likelihood image reconstruction method (Q.Clear) on a BGO PET/CT scanner. Med Phys. (2018) 45(7):3214–22. 10.1002/MP.12986 29782657

[35] Weehaeghe DV Babu S Vocht JD Zürcher NR Chew S Tseng CEJ Moving toward multicenter therapeutic trials in amyotrophic lateral sclerosis: feasibility of data pooling using different translocator protein PET radioligands. J Nucl Med. (2020) 61(11):1621–7. 10.2967/JNUMED.119.241059 32169920 PMC9364895

[36] van der Weijden CWJ Biondetti E Gutmann IW Dijkstra H McKerchar R de Paula Faria D Quantitative myelin imaging with MRI and PET: an overview of techniques and their validation status. Brain. (2023) 146(4):1243–66. 10.1093/brain/awac436 36408715 PMC10115240

[37] Scott CJ Jiao J Melbourne A Burgos N Cash DM Vita ED Reduced acquisition time PET pharmacokinetic modelling using simultaneous ASL–MRI: proof of concept. J Cereb Blood Flow Metab. (2019) 39(12):2419. 10.1177/0271678X18797343 30182792 PMC6891000

[38] Deidda D Karakatsanis NA Robson PM Tsai YJ Efthimiou N Thielemans K Hybrid PET-MR list-mode kernelized expectation maximization reconstruction. Inverse Probl. (2019) 35(4):044001. 10.1088/1361-6420/AB013F

[39] Wadhwa P Thielemans K Efthimiou N Wangerin K Keat N Emond E PET image reconstruction using physical and mathematical modelling for time of flight PET-MR scanners in the STIR library. Methods. (2021) 185:110–9. 10.1016/J.YMETH.2020.01.005 32006678

[40] Gillman AG Quah MH Papoutsellis E Thielemans K Bourgeat P Delplancke C Anatomically-guided deconvolution of PET using directional total variation regularization. *medRxiv* [Preprint]. (2023). p. 2023.04.23.23289004. 10.1101/2023.04.23.23289004

[41] Silva-Rodríguez J Cortés J Rodríguez-Osorio X López-Urdaneta J Pardo-Montero J Aguiar P Iterative structural and functional synergistic resolution recovery (iSFS-RR) applied to PET-MR images in epilepsy. IEEE Trans Nucl Sci. (2016) 63(5):2434–42. 10.1109/TNS.2016.2527826

[42] Rahmim A Qi J Sossi V. Resolution modeling in PET imaging: theory, practice, benefits, and pitfalls. Med Phys. (2013) 40(6):064301. 10.1118/1.4800806 23718620 PMC3663852

[43] Kearney H Miller DH Ciccarelli O. Spinal cord MRI in multiple sclerosis-diagnostic, prognostic and clinical value. Nat Rev Neurol. (2015) 11(6):327–38. 10.1038/nrneurol.2015.80 26009002

